# Echoes of the Month

**Published:** 1924-01

**Authors:** 


### ECHOES OF THE MONTH.

### WHOLEMEAL BREAD FOR ADOLESCENTS.

Speaking on the "Care of the Adolescent," Sir Bruce Porter said that in the early 'teens there is a marked change in the desires for food of both boys and girls, and the boys select weird articles of diet, while the girls tend to become fastidious. This is the important time in which to establish a correct dietary. Bread should be wholemeal, and white bread should be barred from the school as well as the nursery. Much of the flour imported is white flour, as wholemeal flour will not keep. There is no reason why English wheat should not be milled as required, and our children given the chance of really good bread instead of the white rubbish with which they now fill their little stomachs.

### A DANGEROUS INSTRUMENT.

We lived through auto-suggestion, said M. Coué at one of his recent lectures in London. All that we did was done by it. One instance was the crying of a baby in its cradle in order that it might be taken up by its mother. A wise mother left it in the cradle. It was a dangerous instrument, for if used badly it produced bad effects, and it was often used badly. But if a person learnt the danger, it could be avoided. Every idea we had became a reality in the domain of possibility. Although it was not possible to cause any part of our body to grow again if removed, yet it was possible to remove pain and cure unhealthy organs. The idea of sleep created sleep, and the idea of insomnia created insomnia. Some people were deaf, blind or paralysed only because they believed it. It was possible to cure these people. M. Coué gave several instances of such cures. He had treated a young woman who had not been able to see out of one eye for twenty years. She saw immediately after attending one lecture. This was not miraculous, for the patient had lost her sight simultaneously with an illness contracted when two years of age. When cured of the illness, she preserved the habit of not seeing.

### THE BLACK SPOT FOR CANCER.

According to the Medical Officer at Billericay, in Essex, his district has a higher percentage of cancer cases than any other part of the country. The Imperial Cancer Research Campaign have taken note of the statement, but their statistics are not sufficiently complete to put them in a position to make any definite test. For some time the Campaign authorities have had the intention to publish a cancer map of the country, but the production of such a map if it is to be of real value is by no means the simple task it may appear. Take Billericay itself as an example. It may be, as the district Medical Officer says, the blackest spot in the country, but that would be no reason for supposing that there is anything in either the soil or the air that predisposes this district to cancer. On the contrary, the district is rather noted as being particularly healthy, and the medical report suggests that the cause of the abnormal number of cases is that many people go there for health reasons, "some of them suffering from cancer."

### FATIGUE AND SLEEP.

Lecturing on "Fatigue and Sleep," Sir Robert Armstrong Jones said that it was most important that teachers should understand the effects of fatigue, a subject that has only within the last few years been scientifically studied with regard to industry. The view held by some people that fatigue was a normal condition was rather apt to do harm. There were two signs of fatigue—the subjective, such as a feeling of tiredness and a loss of attention, and the objective, which came first. The trouble was that so often the results of fatigue were manifest before a person realised that he was tired. That accounted for many accidents, especially with railway signalmen. This was a dangerous condition to get into, and often resulted in serious mental breakdown. The teacher should note the preliminary signs, such as irritability. The person suffering from fatigue became acutely conscious of sounds which otherwise might pass unnoticed. A closing door seemed to slam, the ticking of a clock sounded loudly, but this state of mental excitement sometimes led to increased effort and output. Discussing the best methods of inducing sleep, Sir Robert said that auto-suggestion was one good way. M. Coué's method was excellent, but a man could overdo it and keep himself awake by his insistence that he was going to sleep. Sir Robert suggested that it was much better to think about other people, and to picture someone else going to sleep. Bedtime should be not later than eleven o'clock.

### TWO NEW DISCOVERIES.

Two new discoveries in medical science are arousing keen interest among medical men. They are anti-microbin for use in cases of pneumonia and rivanol for dealing with peritonitis, lockjaw, etc. The discovery of anti-microbin is the work of Dr. Tomarkin, a Swiss scientist. Its use, it has just been learned, was suggested in the last illness of the ate Pope. It is said to destroy the germ in the blood. This method of treating pneumonia is quite new, and the reports on it are remarkable. The results of the experiments carried out in the Holy Ghost Hospital had been extraordinary. Out of twenty-five pneumonia patients treated, twenty-four were saved by it.

### A DESPISED SENSE.

The sense of smell, says Dr. Harry Robert, far more than hearing or sight, is related in the most intimate way with the unconscious physiological processes of our life. We can turn aside our heads and often refrain from seeing that which we do not wish to see, or focus our gaze on objects that seem desirable. There is nothing to prevent our closing our ears against all the sound that helps to make life difficult. It is not so easy to avoid the smells of the world. They obtrude themselves willy-nilly, and no impression more vitally affects our soul or retains a stronger hold on our unconscious memory than many of the stinks and fragrances which have held our conscious attention perhaps but for a moment. Our receptive olfactory organs are, as one would expect from their earlier development in our evolution, relatively simple affairs. Compared with our eyes and our ears, they are almost primitive in their apparent simplicity. Yet how much of our enjoyment, as well as our health, depends on this almost despised sense! What would our meals be like if they had no flavour? Yet flavour is purely a matter of smell. The sense of smell is our great natural medical officer of health and sanitary inspector; it warns us of the unwholesome, and draws us by æsthetic appeal to that which is pure and desirable.

### SLEEPING SICKNESS.

Professor Kleine, who has been testing the new remedy —Bayer 205—for sleeping sickness in the Union of South Africa, Rhodesia and the Congo, states that it far surpasses atoxyl or any other known remedy. Relapses occurred in only two out of 95 cases. In both cases the third and most serious stage of the disease, in which the nervous system is attacked, had been reached. The remaining cases, which included several of the third stage, were completely cured. Further research will be necessary before a final cure of the disease can be effected in cattle. Healthy animals which had been inoculated with Bayer 205 were, however, protected for several months after entering tsetse-infested areas, and could be used for slaughter.

### AMERICANS DRINK MORE MILK.

Dr. Gerald Leighton, who has recently returned to Scotland from America, where he represented the Scottish Board of Health at the World's Dairy Congress, said, in an interview, that when he landed back in London and stepped into the Strand, the impression he got was that of an orderly peaceful quiet village, in which one might walk with perfect safety and without any risk. They have started recently in the main streets of New York an automatic system of regulating traffic by means of changing coloured lights. When a red light is shown from an elevated platform in the middle of the street the whole traffic is supposed to go up and down the street. When a green light is shown the traffic is supposed to go across the street. Speaking of the purpose of his visit he said that where America is most ahead of Britain is in the successful way in which the trade combines with educational and other authorities in propaganda to educate children and the public as to the value and place of milk in the national diet. "In one district," he said, "the American National Dairy Council, through its dramatic plays, has this year given exhibitions of milk plays and stories before 19,000 people, and 150 meetings have been held at which 25,000 farmers have been addressed. As a result of the Council's activities the total consumption of milk in the States has increased by 16 per cent. in the last two years. The second point from which we can perhaps learn most from American methods is in connection with the great development of machinery used in the distribution of milk and elimination of handling by workers. In some cities it is allowed to be distributed only in bottles, and no 'loose' milk can be sold in the streets."

### LONGER LIVES.

Speaking at a women's meeting at Eltham, Sir Kingsley Wood said that one of the bright spots in our national affairs was that we were never such a healthy nation in our history. There were 600,000 people over 70 years of age, and 60,000 over 85. These results were due to the advance of public sanitation and personal hygiene. Nothing would improve national health more than steady employment and renewed prosperity.

### THE MENACE OF THE RAT.

Writing in the *Western Daily Press*, on "The Harmful Rat," Dr. William Savage says: "I happened, rather over twenty-two years ago, to diagnose the first case of plague recognised in England and Wales since the bacillus had been known. He was a sailor who had come from an Argentine port, where plague was present. On board the ship rats had been dying, and it was his job to throw them overboard. In that way he was no doubt bitten by a plague-infected flea. Six months later we had a second case, and this time infected rats got into the port, and I was able to isolate the bacillus for the first time from an infected rat in this country. Both patients died, but the disease did not spread, partly due to the tremendous war on the rats in the docks. To prevent plague, every endeavour has to be made to prevent rats, especially on ships from plague-infected ports, from landing from the ships. Once they get in the disease may spread widely among the native rats, and the disease extend to man. Some years ago this actually occurred in the East of England, and many native rats (the English brown rat, not the black rat of most of the ships) died of plague."

### LEPERS IN THE UNITED STATES.

Costs of construction have increased 20 per cent. in Louisiana since last spring, when Congress provided $650,000 for the construction of additional buildings at the leper settlement at Carville, Louisiana. The funds, therefore, are sufficient only for the erection of 17 cottages housing 12 lepers each, and the infirmary needed for treatment of the blind and crippled must await further appropriations. There are now 174 lepers at Carville, every bed being filled, the inmates including men and women from nearly every State in the Union. The new buildings authorised will add 204 additional beds, which will be immediately utilised, since there is a waiting list of more than 100 who wish to enter the institution and many other lepers in the United States, aggregating, it is believed, more than 1,000, whom it is desired to segregate as soon as facilities can be provided. One-fourth of the inmates at Carville are totally blind from the disease, and the mutilations, especially of hands and feet, resulting from the disease are forcible reminders of Biblical descriptions.

### CHANGE IN R.A.M.C. BADGE.

The small red Geneva Cross worn on the left sleeve by men of the R.A.M.C. is to disappear. By a decision of the Army Council, this cross, which consisted of red thread worked upon a background of yellow cloth, sewn on to the tunic, will be displaced by a white brassard bearing the red Geneva Cross. The reason for the change is that the old cross was of little practical protection in war. It frequently became obscured by mud and rendered unsuitable for its purpose. Further, largely for these reasons, the badge was unpopular among the personnel of the corps. It is believed that the new white brassard will be of far greater service in distinguishing its wearers to the enemy. It was frequently adopted in the line during the campaigns in France and Flandeis.

### DIMINISHING X-RAY DANGERS.

Details have been published of a new X-rays invention, which, it is claimed, will eliminate all danger to the operator. The invention takes the form of a torch-like tube that can be held in the hand, and by which the rays may be focussed locally without exposing the operator to the deadly beams. It has been perfected by two Dutch medical men, Dr Holst and Dr. Bouwers. Discussing the new invention, the radiologist of a London hospital said it certainly seemed to mark an advance in medical progress, but he doubted whether it would ever be possible entirely to eliminate X-ray dangers. "But the days of X-ray martyrdom are now over. It is a peculiar fact, too, that many radiologists acquire immunity from long association with the rays. It all depends on the bodily condition of the operator. Some people are more susceptible to the rays than others, but personally I have been handling the rays for over twenty years without ill effect."

### NEW TREATMENT FOR SCURVY.

Experiments with a new extract, in tablet form, of watercress, spinach and other green plants called phyllosan, which, it is suggested, may supersede limejuice for the treatment of scurvy, are being made by the Admiralty. Phyllosan, the discovery of Professor E. Buergi, of Berne University, is made from chlorophyll, the vital fluid in plants which bears a close chemical relationship to human blood pigments. Conspicuous success, it is claimed, has attended the Royal Air Force experiments on troops in Iraq suffering from scurvy, malaria and general debility.

### MORTALITY STATISTICS IN GERMANY.

A *Times* correspondent states that while there is enough food in Germany, there is yet considerable want among the population. One may dismiss a good many of the stories that reach England as belonging to the psychosis of charity. Starving people do not habitually fall dead in the Berlin streets, nor do Berlin children die like flies—though I have seen statements in print that they do. But if they did they would have appeared in the death-rate statistics, which give no such indication. In fact, they remain obstinately normal. The first six months of 1923 showed a considerable improvement in public health, especially in regard to epidemics. The infant mortality shows no appreciable advance on 1913. The death-rate for all ages in the first nine months of 1922 was 18·5, compared with 17·3 for the corresponding period of this year, or if infant mortality is excluded, 15·30 last year, as compared with 14·28 this year. The death-rate in 46 large German cities during the third quarter of 1923 was 11·3, as compared with 10·9 in the corresponding quarter of 1922 and 11·5 in 1921, which seems to dispose of the more exaggerated stories.

### A WONDERFUL TRANSITION.

Delivering the inaugural "Lloyd Roberts" lecture, Dr. A. Donald, Chairman of the Medical Board of St. Mary's Hospital, Manchester, traced the progress made in obstetrics and gynæcology during the thirty years between 1880 and 1910. This period, he said, witnessed a transition of a wonderful kind. It was the most eventful period in surgical history since the beginning of knowledge. With the last steps in asepsis, septic peritonitis had been banished from abdominal surgery, except in cases where the germs of infection already existed in the patient's system. Improvement in the technique of operations undoubtedly played a part in the transition, but a much less important one than the thorough adoption of aseptic precautions. Without asepsis technique could have achieved little; with asepsis a technique that was not good might pass muster. Dangers still existed, but we could place the irreducible mortality of a gynæcological surgeon of operating on a large number of abdominal cases at something between 1 and 3 per cent. —the smaller percentage being that of a man who was cautious and the larger that of the man who was willing to operate in cases that were otherwise bound to die.

